# Developing integration among stakeholders in the primary care networks of Singapore: a qualitative study

**DOI:** 10.1186/s12913-022-08165-1

**Published:** 2022-06-15

**Authors:** Shilpa Surendran, Chuan De Foo, David Bruce Matchar, John Pastor Ansah, Josip Car, Gerald Choon Huat Koh

**Affiliations:** 1 Health Systems and Behavioral Sciences Domain, Saw Swee Hock School of Public Health, National University Singapore, Singapore, Singapore; 2 Health Services and Systems Research, Duke-NUS Medical School, Singapore, Singapore; 3 Centre for Population Health Sciences, Lee Kong Chian School of Medicine, Nanyang Technological University, Singapore, Singapore

**Keywords:** Integration, General practitioner, Primary care network, Qualitative research

## Abstract

**Background:**

Integrating healthcare services across and between the different health system levels can be achieved in a few ways; however, examining the social side of integration is essential and challenging. This paper explores the concept of integration perceived by general practitioners (GPs) and primary care network (PCN) representatives from the regional health systems (RHS) in a GP-RHS PCN and their perceived partnership success.

**Methods:**

In this study, we explored three GP-RHS PCNs in Singapore. We used a qualitative research design and, overall, performed 17 semi-structured in-depth interviews with GPs (*n* = 11) and PCN representatives (*n* = 6) from the RHS. All interviews were audiotaped and transcribed verbatim. We conducted thematic analysis to inductively identify themes from the data. Singer's conceptual model of integration types was used as guiding principles to derive relevant and salient themes for integration.

**Results:**

GPs and the RHS perceived the concept of integration through a series of interrelated strategies. Within the normative dimension, a sense of urgency motivated GPs to integrate improvements into their general practice. Participants perceived teamwork and relational climate as appropriate enablers for achieving interpersonal integration in a primary care partnership. While developing a trusted relationship was a perceived success of this partnership across the network, developing camaraderie and gaining knowledge in chronic disease management through the components of functional integration was a perceived success at an individual general practice level. The data also revealed some operational challenges within the structural dimension and some inabilities of the PCN to achieve complete process integration.

**Conclusions:**

Our study points to multi-faceted integration, comprising various forms that need to be manifested at all levels of care to achieve coordinated, seamless, and comprehensive care for patients suffering from chronic conditions. The present iteration of the PCN has been shown to offer integration at a level that warrants praise but still requires structural and process integration improvement.

**Supplementary Information:**

The online version contains supplementary material available at 10.1186/s12913-022-08165-1.

## Background

The global increase in chronic disease burden is necessitating policymakers to restructure their healthcare systems [1]. A key strategy for restructuring is integrating healthcare services within or across the different health system levels [2]. The rationale for integrating healthcare services is twofold. First, patients with chronic conditions require services at different health system levels, and integration aims to facilitate coordination across system levels [3]. Second, traditionally health systems are organised around diseases and organ systems, whereas in an integrated approach, services are organised around patient needs, which can be similar regardless of the specific medical condition. Third, integration can reduce the replication of services across different medical specialities [3]. With the notion that primary care with sufficient capacity and capability is crucial to care integration, the Singapore Ministry of Health (MOH) facilitated the development of the Primary Care Network (PCN) to serve as a vehicle for addressing the increasing chronic disease burden [4–6].

A PCN is a network of general practitioners (GPs) supported by nurses and care coordinators that provides holistic care for patients with chronic conditions [6]. The MOH funds the PCN to hire project managers, nurse counsellors and care coordinators to provide team-based care [6]. Currently, there are two types of PCN models in Singapore, the GP-Driven PCN and the GP-Regional Health Systems (RHS) partnership PCN [6]. An RHS is a geographical cluster led by a public hospital working closely with primary care providers, community hospitals, nursing homes, home care and day rehabilitation providers to foster integrated care within the region [7]. Of the 10 PCNs established in Singapore since 2018, three are GP-RHS PCNs [6].

The GP-RHS PCN is a public–private partnership between GPs and one of the RHS [6]. As these stakeholders often have different views, interests, and objectives, integrating services between them is often difficult to accomplish [8]. Moreover, lack of trust and commitment, ineffective leadership, and communication are other barriers to integrating services across the different health system levels and organisations [8–10].

Integration across the different health system levels and entities can be achieved in a few ways. Structural, functional, and clinical types of integration are the common modes through which healthcare services integration occur. However, a recent study by Singer et al. [11] has highlighted the importance of examining the social side of integration. Firstly, when integrating healthcare services, stakeholders must collaborate in new ways. Secondly, consistent and coordinated approaches to working are more organically adopted when stakeholders share a positive relationship. Therefore, it is imperative to understand the social features pertaining to stakeholder perceptions and underlying beliefs and how they interact to foster integration of healthcare services within and beyond the different health system levels in a coordinated manner.

Interpersonal and normative types of integration describe the social features closely related to stakeholders’ beliefs and behaviour [11]. However, only a handful of studies [12–14] have examined them in healthcare contexts since the concept is challenging to investigate [15–17]. Therefore, this study’s objective is to explore the concept of integration among GPs and the PCN representatives from the RHS in a GP-RHS PCN and their perceived partnership success. This study is central and timely as internationally, countries are moving towards implementing PCNs, which are the building blocks of integrated care systems [18]. Therefore, demonstrating how stakeholders at the different health system levels develop integration will offer valuable insight and evidence-based approaches for researchers and policymakers working on integrated care systems globally.

## Methods

### Theoretical framework

We used Singer et al.’s [11] conceptual model of integration types to study the concept of integration. Integrations, such as structural, functional, normative, interpersonal and process, happen through a range of inter-related integration efforts. Integration is not fixed but rather a fluid process underpinned by the dynamic interplay stakeholders place on the various interactions they have with each other. The conceptual model describes five types of integration at different health system levels. These are:

Structural integration — refers to capital assets, operational, financial, or legal ties and governance mechanisms such as ownership which are all bounded in contractual arrangements within and between organisations.

Functional integration — refers to formal, written policies and protocols for activities which are under the leaders’ direct control and that support coordination, accountability and decision making between organisations and individuals.

Normative integration — refers to sharing a common culture such as shared vision and mission, collective attitude, sense of urgency, and various forms of leadership and exhibiting a specific culture of integration such as coordination, communication, and continuity of care across various organisations.

Interpersonal integration — refers to the extent of teamwork or collaboration among healthcare professionals and nonprofessional caregivers and patients within and between organisations.

Process integration — refers to planned processes or implemented procedures that are explicitly intended to integrate healthcare services across people, functions, and operating units in a single coordinated approach.

### Design and setting

This study is reported using the consolidated criteria for reporting qualitative research (COREQ) guidelines (see Additional file 1) [19]. In this study, we explored three GP-RHS PCNs. In a GP-RHS PCN partnership, there are two leaders. The clinical leader is a GP, while the administrative leadership role is steered by the RHS with whom the GPs have partnered. The size of the network ranges from 72 to 120 general practices [6]. We used a qualitative research design and, overall, performed 17 semi-structured in-depth interviews with GPs (*n* = 11) and PCN representatives (*n* = 6) from the RHS between March 2019 and October 2021 to explore the concept of integration and their perceived partnership success.

### Sampling strategy and data collection

We used a purposive maximum variation sampling approach to recruit participants to obtain a spectrum of qualitative data to construct a holistic understanding of the GP-RHS relationship from various angles [20]. Participants were purposively selected based on their position and expertise within the GP-RHS PCN. “Position” is referred to participants’ position within the PCN, i.e., administrative lead, manager, clinical lead, and primary care coordinator and "expertise” is referred to their specific responsibilities within the PCN (Table 1). A list of general practices participating in PCNs is available on a publicly accessible government-run website designated for the same. Based on this list, we contacted the general practices via telephone or email in sequential order and approached the GP in charge of the practice to explain the objectives of our study. After the initial contact, an official participant information sheet and consent form was sent to the participants. Written informed consent was obtained from all participants prior to commencing the in-depth interviews. None of the participants invited refused to participate in this study.

**Table 1:** Sociodemographic characteristics of participants (*n* = 17)

Participant ID	Age in years	Gender	Position within the PCN	Specific job in the PCN	Years of experience in primary care
RHS 01	49	Female	Manager	Administrative support	22
RHS 02	46	Female	Administrative lead	Administrative support, Fund management, CME curriculum development	8
RHS 03	53	Female	Administrative lead	Administrative support, Fund management, CME curriculum development	25
RHS 04	41	Female	Administrator	Administrative support	10
RHS 05	30	Female	Assistant manager	Administrative support	5
RHS 06	30	Female	Primary care coordinator	Care coordination	3
GP 01	64	Male	General practitioner	Collect chronic disease care indicators	35
GP 02	56	Female	General practitioner	Collect chronic disease care indicators	25
GP 03	40	Male	General practitioner	Collect chronic disease care indicators	12
GP 04	72	Male	General practitioner	Collect chronic disease care indicators	40
GP 05	41	Male	General practitioner	Collect chronic disease care indicators	15
GP 06	55	Male	Clinical lead	Fund management, CME curriculum development, resolve problems faced by the GPs	30
GP 07	49	Male	Clinical lead	Fund management, CME curriculum development, resolve problems faced by the GPs	20
GP 08	39	Male	Clinical sub-lead	Fund management, CME curriculum development, resolve problems faced by the GPs	8
GP 09	59	Female	General practitioner	Collect chronic disease care indicators	27
GP 10	38	Male	General practitioner	Collect chronic disease care indicators	5
GP 11	Not available	Male	Clinical lead	Fund management, CME curriculum development, resolve problems faced by the GPs	10

*RHS* Regional Health Systems, *GP* General practitioner, *PCN* Primary Care Network, *CME* Continuous Medical Education

The sample size was determined based on data saturation. Data saturation was decided by the study team members based on reaching thematic saturation, where no new themes could be identified from the interview data (see Additional file 2). We reached data saturation by the 17^th^ interview. Data were collected using an interview guide. One author conducted the interviews in English, either face-to-face at the participants’ workplace or via a video conferencing platform, and another author took detailed field notes. Interviews were audiotaped and lasted 60 to 80 min on average. All participants approached agreed to participate.

### Data analysis

Interviews were transcribed verbatim, and we conducted thematic analysis to inductively identify themes from the transcripts [21]. Two authors coded the transcripts using QSR NVivo 12 software [22]. The codes were then organised into sub-themes and themes derived from the data with Singer et al.’s [11] conceptual model of integration types as a thematic guide.

To enhance the rigour of the analytical process, three authors discussed codes and themes in regular team meetings to enhance reflexivity and challenge interpretations. Thematic saturation was achieved when analysis revealed no new codes and themes [21]. Each quote includes either GP or RHS, followed by a participant number (GP01/RHS01).

To enhance the study's trustworthiness, firstly, the interviewer and the note taker acknowledged their position as a research team member with the participants to mitigate any preconceived bias [23]. Secondly, the interviewer wrote memos to document her reflections and any emerging themes that were later interpreted to express the types of integration. Lastly, member checking was performed with a few participants in the final data analysis stage to verify their perspectives' accurate representation and validate our interpretations of the data [24].

## Results

We conducted 17 semi-structured in-depth interviews with GPs (*n* = 11) and PCN representatives (*n* = 6) from the RHS. Their average age was 48 years (range 30–72 years), and their average years of experience in primary care was 21 years (range 8–40 years) (Table 1). Table 2 summarises the coding scheme. Themes and subthemes are described in detail below. Data saturation was reached at the 17^th^ interview.

**Table 2:** Coding scheme with count and percentage [n (%)] of codes

Theme	Subtheme	Code
Structural integration	Legal ties	Contract details stakeholders’ roles and responsibilities (13/17, 76%)
	Operational ties and problems	Mandatory data entry challenging (10/11, 90%)
		Coordination issues due to multiple clinic management system vendors (2/6, 33%)
		Coordination issues in deploying ancillary services (2/6, 33%)
		Lack of personal attention (3/11, 27%)
		Regional division of the network helps to build relationships, better coordination, and share the workload among the GP leads (4/6, 67%)
Functional integration	Protocols for activities	Joint decision-making for developing protocols for fund management and continuous medical education curriculum development (15/17, 88%)
		GPs decide how to utilise disbursed care plus fee (11/17, 65%)
		Gain knowledge on chronic disease management (11/11, 100%)
Normative integration	Shared vision	Recruit GPs with an interest in chronic disease management (4/6, 67%)
		Early to comment of PCN’s effectiveness in improving chronic disease care since its functions were disrupted due to the coronavirus infectious disease 2019 pandemic (4/17, 27%)
	Sense of urgency	Adopted clinic management system (11/11, 100%)
		Early to comment of PCN’s effectiveness since Ministry of Health constantly updates the care indicators for data collection. (1/17, 5%)
	Culture of communication	Two-way communication process (9/11, 81%)
		Transparent communication (11/11, 100%)
Interpersonal integration	Teamwork	Administrative and social support (17/17, 100%)
		GP lead donated remuneration (4/17, 24%)
	Relational climate	Solve problems collaboratively (6/6, 100%)
		Trusting relationship (17/17, 100%)
		Reservations about raising their concerns (6/11, 55%)
		No differences in opinion (17/17,100%)
		Camaraderie among the GPs (11/11, 100%)
Process integration	Incomplete closed referral loops	Patients not discharged back to referring GP (2/11, 18%)

*GP* General practitioner, *PCN* Primary Care Network

### Theme 1: structural integration

#### Subtheme 1.1: legal ties

GPs sign a legal contract with the RHS they have partnered with before joining the network. They also get a copy of their signed contract for filing purposes. According to most of the participants (13/17, 76%), the contract stipulates the shared roles and responsibilities of the members and the PCN goals for clarity purposes. The contract also states the RHS's actions if GPs displayed negative behaviours or actions. One participant stated:

“We have a contract with the individual general practice which spells out all the requirements that they (GPs) need to do. The rules and responsibilities are clearly defined. If we find out that anyone does not follow this within our network, they (GPs) do anything which affects the image of the RHS, we will investigate and if needed terminate the general practice.” (RHS 01).

#### Sub-theme 1.2: operational ties and problems

Under PCN, it is mandatory to collect patients’ data for chronic disease indicators. However, the majority of GPs (10/11, 90%) found this compulsory data collection and entry challenging due to their clinical duties. Despite this, they described how their vision to improve care for patients with chronic diseases helped them in setting priorities. For example, some GPs sought their clinic assistants’ help, and others worked overtime to complete the data entry.

Large network size posed some operational challenges to the RHS across the network. For instance, a few RHS participants (2/6, 33%) raised issues in coordination because they had to liaise with multiple clinic management system vendors as GPs commonly use different brands of clinic management systems. Another operational challenge faced by the RHS was the difficulty in deploying shared ancillary services such as diabetic retinal photography and foot screening to all general practices in a coordinated approach. As a result, a few GPs (3/11, 27%) perceived a lack of personal attention from the RHS at an individual general practice level. One participants stated:

“…I was hoping more for personal care like the RHS staff or the nurse to come down and help me to point out where I missed, but I don't get that. I think too big (network size) also has got its own problem….” (GP 01).

Therefore, to tackle the operational challenges in a way that is satisfactory for both parties, more than half of the RHS participants (4/6, 67%) reported further dividing their network into smaller geographical regions or having plans to do so, where each region is led by a GP lead and PCN representatives from the RHS. In addition to building relationships and strengthening coordination, sharing workload among the GP leads were the advantages identified across the network if the network is divided into smaller geographical regions. One participant stated:

“…if we divide the PCN into regions, more GP leads can shoulder the responsibility. Moreover, we can serve the population better by delivering the ancillary services in a more effectively and coordinated manner” (RHS 03).

### Theme 2: functional integration

#### Subtheme 2.1: protocols for activities

According to most participants (15/17, 88%), protocol developments for activities such as fund management and continuous medical education curriculum development (CME) are jointly decided by the exco committee and a few GPs. Exco committee comprise GP leads, PCN representatives from the RHS and with/without a few GPs. Exco members are the primary decision-makers on how to utilise the funds provided by the MOH to run the PCN’s daily operations. They follow the guidelines stated in the protocol when doing so. On the other hand, most of the participants (11/17, 65%) stated that GPs have the freedom to allocate the disbursed care plus fees (an incentive of SGD 100 for extended consultation time [4]) to either subsidise medicines or investigations for their patients or pay bonuses to their clinic assistants.

Protocols for CME curriculum development stipulate chronic disease management must be the focus during the PCN quarterly meetings and the topics must be prepared and scheduled a few months in advance. One participant stated:

“We will work with our specialists to prepare a list of topics important for chronic disease management, … sometimes GPs will also flag out to us on the topics which they think are relevant. The list of topics is prepared in advance and meetings are already scheduled.” (RHS 04).

### Theme 3: normative integration

#### Sub-theme 3.1: shared vision

The RHS recruited like-minded GPs whose vision was to manage patients with chronic conditions according to most of the RHS participants (4/6, 67%). Similarly, all the GPs mentioned that their interest to manage complex chronic diseases was the main reason for joining the network. Together they created shared expectations for the PCN, sensemaking of the PCN’s goals and objectives and built consensus on what to do. One participant stated:

“I joined PCN with a vision to improve care for patients with chronic diseases.” (GP 04).

Nevertheless, according to a few participants (4/17, 24%), it is still too early to comment on the effectiveness of the PCN in improving chronic disease care due to the massive disruption faced to the operations of the PCN attributed to the coronavirus infectious disease 2019 (COVID-19) pandemic.

#### Sub-theme 3.2: sense of urgency

All GPs understood that to manage patients with chronic conditions, they had to collaborate with the RHS to make changes at the operational level. For this, at an individual general practice level, all the GPs systematically either adopted an electronic clinic management system or upgraded their existing clinic management system to collect patients’ care indicators. One participant stated:

“For my patients I used to trend their results on paper (patient card). I did not use an electronic clinic management system. Now in PCN, I understand its need and I have bought one software…” (GP 02).

Nevertheless, according to one participant, it is still too early to comment on the effectiveness of the PCN on improving patients’ clinical outcomes. This is because the MOH constantly updates the care indicators for data collection. Hence, the clinic management system vendors could not keep up with constant change, and as a result, the data collection was incomplete.

#### Sub-theme 3.3: culture of communication

Participants perceived the two-way communication process and transparency as key components of a culture of communication and as an essential concept affecting the partnership’s success across the network. Majority of the GPs (9/11, 81%) stated that the RHS promoted and maintained a two-way communication channel between the GPs and the RHS. For example, GPs were encouraged to share their opinions during the PCN quarterly meetings and the RHS would share their constraints and perspectives for the same if they had any. Furthermore, the RHS was receptive and responsive to GPs’ queries and feedback. These frequent two-way interactions enabled both the RHS and the GPs to address issues collaboratively and helped develop a trusted relationship.

Transparency in disclosing financial data by the RHS has helped foster a trusted relationship with the GPs across the network, which is deemed as a perceived success of this partnership according to all the participants. Similarly, routine interactions among GPs during the PCN quarterly meetings have helped them build camaraderie at an individual general practice level, another perceived success of this partnership. One participant stated:

“We are very transparent to all members about whatever funding MOH gives us. We show in the slide all the funding we get to our members and tell them how we will distribute this fund, make use of it, and what initiatives we will do in the next five years […] we have developed trust with our members (GPs). A trusted relationship is easy to break without being transparent.” (RHS 01).

### Theme 4: interpersonal integration

#### Sub-theme 4.1: teamwork

All GPs had positive attitudes about teamwork, reflecting high levels of teamwork amongst GP colleagues within the network. For instance, GPs would inform fellow GPs from where they could purchase medicines at low prices. Similarly, the RHS went the extra mile by providing additional administrative and social support to the GPs. For example, the RHS aided elderly GPs in using computers by ‘hand-holding’ them. Moreover, the RHS would visit general practices to resolve issues between the clinic assistants and their GPs. Obtaining administrative and social support was perceived by the GPs as a success of this partnership at an individual general practice level and across the network. One participant stated:

"Some GPs are scared of touching the computer because they are not tech-savvy. […] So, we give them a lot of assurance and hand-holding. A 70-year-old GP never used the computer […], so we spend months going down many times […], and we do this until GPs feel convenient to use the system […]" (RHS 01).

A few participants (4/17, 24%) praised their GP lead for being an excellent team player by donating her remuneration to the PCN to improve its operational effectiveness. One participant stated:

"Clinical leader is so gracious; she refused to take the money, but she'll put it back to the PCN to run the programmes.” (GP 02).

#### Sub-theme 4.2: relational climate

The PCN quarterly meetings facilitated the GPs and the RHS to nurture strong trusting relationships in many ways. First, the quarterly meetings facilitated a greater ease for the RHS in partnering GPs to testbed novel programmes at the primary care level and served as a platform to understand the problems faced by the GPs. This helped them to solve problems collaboratively and was attributed to the partnership’s success across the network by all the RHS participants. Some GPs (6/11, 55%) indicated having reservations about raising their concerns during the PCN quarterly meetings. Generally, all participants expressed an innate desire to cooperate, and there were no differences in opinion where they had to negotiate. Building a strong relationship was a perceived success of this partnership across the network by both parties. One participant stated:

“… earlier, there was no strong relationship with the GPs. PCN helps us strengthen the relationship because the senior management comes and meet up with the GPs in the quarterly meetings and talk to them. Previously nothing like that has been possible. […] So, I think relationship building is definitely a big plus.” (RHS 02).

Second, at the quarterly meetings, GPs present condensed or detailed case histories to elicit discussions about handling clinical problems. GPs ask questions, make comments, and share anecdotes triggered by the case discussions. The informal atmosphere in the meetings also allowed proposals and ideas for improving the functioning of the PCN. According to all the participants, camaraderie and gaining knowledge on chronic disease management is a perceived success of this partnership at an individual general practice level. Gaining knowledge in chronic disease management improved their confidence in treating patients with chronic diseases. Moreover, they also mentioned having an advantage over non-affiliated GPs to receive early specialists’ appointments in the RHS tertiary hospitals for their patients.

### Theme 5: process integration

#### Sub-theme 5.1: inadequate closed referral loop

A few GPs (2/11, 18%) mentioned that when they referred their patients to the hospitals, some tertiary care doctors would not discharge the patients back to the referring GPs. Instead, they would discharge the patients to other GPs or primary care centres. They mentioned highlighting this problem to their PCN representatives from the RHS. On the other hand, one RHS participant attributed the lack of coordination to increased turnaround of junior doctors and their lack of awareness of the discharging procedures. One participant stated:

“All along they (RHS) will say they will do it and refer back to the referring doctor. I was a bit upset as I said because they refer to another clinic which I think is not really fair to us. I mean, we always say when we refer to you, you refer back to us what. That is the usual.” (GP 01).

## Discussion

Our study suggests that the GPs and the RHS perceived the concept of integration through a series of interrelated strategies. Within the normative dimension, sense of urgency inspired GPs to integrate improvements into their general practice. Participants perceived the key features of interpersonal integration as appropriate enablers for achieving integration in a primary care partnership. While developing a trusted relationship through interpersonal integration was a perceived success of this partnership across the network, gaining knowledge in chronic disease management through the components of functional integration was a perceived success at an individual general practice level. The data also revealed some operational challenges within the structural dimension and some inabilities of the PCN to achieve complete process integration.

### Interpersonal integration

Teamwork was perceived as an appropriate enabler for achieving interpersonal integration. Providing administrative support by the RHS allows GPs to allocate more time to their increasing clinical workload [25]. However, it is easy for GPs to develop ‘administrator dependency’ over time. The MOH has decided to support the PCN scheme for five years, i.e., until 2023, after which the future of PCN is uncertain [6]. If the GP-RHS PCN undergoes privatisation, GPs will be forced to helm the administrative position without being sufficiently prepared for the task. This can lead to frustration and operational ineffectiveness of the network. Moreover, GPs might feel a strain taking on dual capacities, i.e., as an administrative leader of the network and a provider in their general practice [4]. Therefore, to effectively integrate GPs into managerial roles and decrease administrator dependency, the RHS should take a step back, delegate administrative tasks to the GPs and create a culture of responsibility. Additionally, the managerial competencies of GPs can be nurtured by incorporating a course on management into CME curriculum and by adequately compensating GP administrators for their full-time equivalent required to run the PCNs [4].

### Relationship between structural and normative integration

A shared vision among professionals and organisations that engage in integrated services is widely considered an integral element of a successful partnership [26]. Our study supports this because the GPs and the RHS had already developed a vision during the development phase of the PCN when they were applying for funding to MOH through a joint proposal [4].

A sense of urgency for chronic disease management encouraged GPs to collaborate with the RHS for data collection. Moreover, legal, and operational ties also pushed the GPs to collaborate with the RHS for data collection. However, overloading the GPs with data entry was identified as a problem. General practices undergo numerous changes when integrating healthcare services and experience an acceleration trap [8, 27]. Due to the accelerated pace of change, it is highly likely that the GPs become exhausted, affecting their relationship with the RHS and the partnership's success [27]. A recent study by Surendran et al. [8] found that relational imbalances between the GPs and the RHS affected the success of their primary care partnership. Therefore, studies investigating the effect of acceleration trap in a clinical setting are critical to developing new population-level integrated care programmes that can achieve the intended outcomes. Moreover, establishing role clarity with explicit descriptions of the responsibilities of the stakeholders could be beneficial for normative integration [8, 13].

### Relationship between functional, interpersonal, and normative integration

Joint decision-making processes among stakeholders when developing protocols for activities can result in transparent communication. Transparent communication will help develop trust-based relationships among the stakeholders, which is essential for nurturing the collaborative ethos necessary for successful integrated care programmes, and our study strengthens this empirical evidence [8, 28]. This implies that more functional integration is associated with more normative integration leading to more interpersonal integration. However, it is possible that the GPs and the RHS might have already developed a trusting relationship through other programmes, and hence re-fostering transparency is not needed [29–31]. But in times of ambiguities, transparent communication will guide unsure GPs on which RHS to trust as a partner or continue its relationship with [31]. Moreover, adopting transparency will increase the reputation and positive image of the stakeholders and create an opportunity to acquire consensus and appreciation from all the stakeholders in the partnership [32, 33]. Our study findings showed no issues where the GPs and the RHS had to negotiate to reach a consensus. However, disclosing information is not without its risks. Stakeholders must be aware that the information disclosed can be used against them, which might counteract normative and interpersonal integration [33]. For example, when communicating financial information and being made aware of the funding pool, GPs might put forward conflicting demands that are difficult for the RHS to fulfil, thereby affecting their relational capital.

### Limitations and strength

Firstly, although PCN was formally established in 2018, its functioning was disrupted by the COVID-19 pandemic. Therefore, we need to interpret the results of this study as originating from an early phase of PCN’s implementation. Secondly, since integration evolves, a longitudinal perspective with follow up interviews is recommended [34]. Lastly, regarding the transferability of results, caution should be taken when extending the findings to PCNs solely run by GPs. This is because different PCN models operate in different contexts and environments. Therefore, the findings of this study will be relevant for PCN models similar to the one explored here, i.e., a public–private primary care partnership. The strength of this study is that we captured divergence in perspectives through in-depth interviews with a maximum variation sampling strategy.

### Implications

This study provides valuable information on developing effective and deployable strategies for integration in a primary care context. Furthermore, the structural dimension also provides information on the disadvantages of a large network size. We propose that problems with large network size (e.g., lack of personal attention) can be circumvented by creating sub-teams, each having a GP leader reporting to the overall PCN leaders, to foster a culture of coordination and communication. However, in small teams, the risk for relational imbalances cannot be ruled out since different personalities are more prone to clashes. Therefore, teams should develop a flexible mindset away from fixed thinking and track the network's facilitators and barriers while working towards the common vision of enhanced chronic disease management [35]. The different ways of using the care plus fee i.e., a flexibility in functional integration, create a lack of uniformity within the network. However, this flexibility is desired to develop and maintain interpersonal integration in a public–private partnership [8]. Future research should use objective data to examine how the integration strategies developed in this study relate to the impact of a partnership on patient health and cost-related outcomes [34].

## Conclusion

Our study points to multi-faceted integration, comprising various forms that need to be manifested at all levels of care to achieve coordinated, seamless, and comprehensive care for patients suffering from chronic conditions. The present iteration of the PCN has been shown to offer integration at a level that warrants praise but still requires structural and process integration improvement. Increasingly, and as seen through the COVID-19 pandemic, primary care has become the backbone of the healthcare system. Therefore, the PCN needs to explore the incorporation of infectious diseases protocols as an additional module into their operations to leverage on pre-existing relationships and integrative processes to manage a wider range of diseases going into the future.

## Supplementary Information

**Additional file 1.** Consolidated criteria for reporting qualitative studies (COREQ): 32-item checklist.

**Additional file 2.** Interview guide.

## Data Availability

The datasets generated during and/or analysed during the current study are not publicly available due to participants’ privacy concerns. Readers who wish to gain access to the data can write to the corresponding author; data may be granted upon reasonable request.

## Abbreviations

MOH: Ministry of Health
PCN: Primary Care Network
GPs: General Practitioners
RHS: Regional Health Systems
CME: Continuous Medical Education
COVID-19: Coronavirus Infectious Disease 2019

## References

[1] Borgermans L, Marchal Y, Busetto L (2017). How to improve integrated care for people with chronic conditions: key findings from EU FP-7 project INTEGRATE and beyond. Int J Integr Care.

[2] Nurjono M, Yoong J, Yap P (2018). Implementation of integrated care in Singapore: a complex adaptive system perspective. Int J Integr Care.

[3] PAHO (2013). Innovative care for chronic conditions: organizing and delivering high quality care for chronic noncommunicable diseases in the Americas.

[4] Foo CD, Surendran S, Tam CH (2021). Perceived facilitators and barriers to chronic disease management in primary care networks of Singapore: a qualitative study. BMJ Open.

[5] Foo CD, Surendran S, Matchar DB (2021). Primary care networks and starfield’s 4Cs: a case for enhanced chronic disease management. Int J Environ Res Public Health.

[6] MOH Singapore. Primary care networks for better patient care in the community, https://www.moh.gov.sg/news-highlights/details/primary-care-networks-for-better-patient-care-in-the-community (accessed 29 Oct 2021).

[7] Improving accessibility, quality and affordability for tomorrow’s challenges, https://www.moh.gov.sg/news-highlights/details/moh-2012-committee-of-supply-speech-healthcare-2020-improving-accessibility-quality-and-affordability-for-tomorrow-s-challenges-(part-1-of-2) (accessed 29 Oct 2021).

[8] Surendran S, Foo CD, Tam CH (2021). The missed opportunity of patient-centered medical homes to thrive in an asian context. Int J Environ Res Public Health.

[9] Andersson J, Ahgren B, Axelsson SB (2011). Organizational approaches to collaboration in vocational rehabilitation-an international literature review. Int J Integr Care.

[10] Kozlowska O, Lumb A, Tan GD (2018). Barriers and facilitators to integrating primary and specialist healthcare in the United Kingdom: a narrative literature review. Future Healthc J.

[11] Singer SJ, Kerrissey M, Friedberg M (2020). A comprehensive theory of integration. Med Care Res Rev.

[12] Oksavik JD, Aarseth T, Solbjør M (2021). ‘What matters to you?’ normative integration of an intervention to promote participation of older patients with multi-morbidity - a qualitative case study. BMC Health Serv Res.

[13] Poulsen RM, Pii KH, Bültmann U (2019). Developing normative integration among professionals in an intersectoral collaboration: a multi-method investigation of an integrated intervention for people on sick leave due to common mental disorders. Int J Integr Care.

[14] Dickinson A, Joos S (2021). Barriers to integration of primary care into emergency care: experiences in Germany. Int J Integr Care.

[15] Valentijn PP, Schepman SM, Opheij W (2013). Understanding integrated care: a comprehensive conceptual framework based on the integrative functions of primary care. Int J Integr Care.

[16] Strandberg-Larsen M, Krasnik A (2009). Measurement of integrated healthcare delivery: a systematic review of methods and future research directions. Int J Integr Care.

[17] Goodwin N (2013). Taking integrated care forward: the need for shared values. Int J Integr Care.

[18] NHS England. Primary Care Networks: The building blocks of an integrated care system, https://www.england.nhs.uk/gp/case-studies/primary-care-networks-the-building-blocks-of-an-ics/. Accessed 1 Nov 2021.

[19] Tong A, Sainsbury P, Craig J (2007). Consolidated criteria for reporting qualitative research (COREQ): a 32-item checklist for interviews and focus groups. Int J Qual Health Care.

[20] Benoot C, Hannes K, Bilsen J (2016). The use of purposeful sampling in a qualitative evidence synthesis: a worked example on sexual adjustment to a cancer trajectory. BMC Med Res Methodol.

[21] Braun V, Clarke V (2006). Using thematic analysis in psychology. Qual Res Psychol.

[22] Qualitative Data Analysis Software | NVivo, https://www.qsrinternational.com/nvivo-qualitative-data-analysis-software/home (accessed 29 Oct 2021).

[23] Lincoln YS, Guba EG (1985). Naturalistic inquiry.

[24] Goldblatt H, Karnieli-Miller O, Neumann M (2011). Sharing qualitative research findings with participants: study experiences of methodological and ethical dilemmas. Patient Educ Couns.

[25] Willis M, Duckworth P, Coulter A (2020). Qualitative and quantitative approach to assess of the potential for automating administrative tasks in general practice. BMJ Open.

[26] Kaehne A (2020). Sharing a vision. Do participants in integrated care programmes have the same goals and objectives?. Health Serv Manage Res.

[27] Martin J, McCormack B, Fitzsimons D (2014). The importance of inspiring a shared vision. Int J Prac Dev.

[28] Frerichs L, Kim M, Dave G (2017). Stakeholder perspectives on creating and maintaining trust in community-academic research partnerships. Health Educ Behav.

[29] Yeo SQ, Harris M, Majeed FA (2012). Integrated care for diabetes-a Singapore approach. Int J Integr Care.

[30] Nurjono M, Shrestha P, Ang IYH (2020). Shifting care from hospital to community, a strategy to integrate care in Singapore: process evaluation of implementation fidelity. BMC Health Serv Res.

[31] Montgomery T, Berns JS, Braddock CH (2020). Transparency as a trust-building practice in physician relationships with patients. JAMA.

[32] Creixans-Tenas J, Gallardo-Vázquez D, Arimany-Serrat N (2020). Social responsibility, communication and financial data of hospitals: a structural modelling approach in a sustainability scope. Sustainability.

[33] Dincer H, Yuksel S (2019). Global issues in financial communication and investment decision making.

[34] Valentijn PP, Vrijhoef HJM, Ruwaard D (2015). Exploring the success of an integrated primary care partnership: a longitudinal study of collaboration processes. BMC Health Serv Res.

[35] Hysong SJ, Amspoker AB, Hughes AM (2019). Impact of team configuration and team stability on primary care quality. Implement Sci.

