# Supplementary material for: Developing integration among stakeholders in the primary care networks of Singapore: a qualitative study
Source: BMC Health Serv Res. 2022 Jun 15;22:782. doi: 10.1186/s12913-022-08165-1 (PMC9198200; doi:10.1186/s12913-022-08165-1)
Supplement: Supplementary file 2 — Additional file 2. Interview guide. [file 12913_2022_8165_MOESM2_ESM.docx]

Additional file 2: Interview Guide

| **Section I**: Introductory questions |
| --- |
| 1. To start, can you tell me more about yourself?  - Can you share more about your professional background? - Current role/roles and job scope? - Can you share about your educational background ? |
| **Section II**: About PCN |
| 1. How does the functioning of the PCN facilitate primary care in Singapore? 2. Can you describe the organisation/s and capacities of the PCN? 3. How did the current primary care schemes such as PCN come about? 4. What do you think are the major challenges of the implementation of the PCN? Why? 5. What facilitated implementation of PCN? Why? 6. Can you comment about the specific efforts to influence the implementation PCN? 7. What factors have promoted or inhibited any of the efforts you mentioned? |
| Closing questions and remarks |
| 1. Before we end, do you have any final thoughts to share? 2. We have come to the end of the interview. Do you have any other questions? |
